# Analysis of urgent follow up visits and complications after intravitreal injections: a retrospective cohort study

**DOI:** 10.1186/s40942-021-00358-w

**Published:** 2022-01-18

**Authors:** Alexander Miller, Matthew A. Wilneff, Andrew Yazji, Emily Petrinec, Michael Carbone, Chase Miller, Christina McCrossin, Richard Donkor, David G. Miller

**Affiliations:** 1 Northeastern Ohio Medical University, Rootstown, OH USA; 2 Florida Atlantic University Charles E. Schmidt College of Medicine, Boca Raton, FL USA; 3 Retina Associates of Cleveland Inc, 24075 Commerce Park, Cleveland, OH USA; 4 Ohio University Heritage College of Osteopathic Medicine, Athens, OH USA; 5 University Hospital - Mason Eye Clinic, Columbia, MO USA

## Abstract

**Background:**

Intravitreal injections (IVIs), a common treatment in ophthalmology, result in acute complications and urgent follow-up visits causing significant burden to both patient and physician. We evaluated the incidence of acute complications following IVIs which occurred within seven days of injection.

**Methods:**

A retrospective cohort study conducted at a private retinal practice, in Cleveland, Ohio. Using the practice management software database, we examined 73,286 injections of patients with unscheduled or urgent visits within 7 days of an injection from August 1st,2018 to August 1st,2020. Data collected included: age, gender, eye, medication injected, diagnosis, reason for urgent follow-up, time between injection and urgent follow-up, and type of anesthesia administered. Data was analyzed using SPSS v.28 (SPSS Inc., Chicago IL).

**Results:**

Study included 73,286 injections, with 441 injections (n = 441) resulting in urgent follow-up visits (0.60%). Mean patient age was 72.1 (± 30.4) years, with 187 male (42.4%) and 254 female (57.6%) patients. IVI medications included: aflibercept (60.3%), ranibizumab (22.4%), bevacizumab (13.4%), dexamethasone intravitreal implant (2%), triamcinolone acetonide (1.6%) brolucizumab (1.59%), fluocinolone acetonide intravitreal implant 0.19 mg (0.2%), and fluocinolone acetonide intravitreal implant 0.18 mg (0.03%) (Table 1). Medications associated with urgent visits included: aflibercept (42.9%), bevacizumab (37.4%), ranibizumab (7.9%), dexamethasone intravitreal implant (6.8%), brolucizumab (2.7%), and triamcinolone acetonide (2.3%) (Table 2). Days between injection and urgent follow-up was on average 3.96 ± 2.14 days. Urgent follow-ups included blurred vision in 164 patients (37.2% of urgent visits), flashes, floaters or posterior vitreous detachment (PVD) in 55 (12.5%), pain in 42 (9.5%), 43 (9.8%) corneal abrasions, 33 (7.5%) subconjunctival hemorrhages, corneal dryness or foreign body sensation in 30 (6.6%), endophthalmitis in 20 (4.5%), 18 (4.1%)vitreous hemorrhages, iritis or uveitis in 11 (2.5%), miscellaneous complications in 9 (2.0%), 7 (1.6%) elevated intraocular pressures, choroidal neovascular membrane in 4 (0.9%), 4 (0.9%) retinal detachments or tears, and 2 (0.45%) traumatic cataracts (Table 3).

**Conclusion:**

IVIs resulted in 0.60% urgent/unscheduled follow-up visits within 7 days of injection. Most common causes were blurred vision and symptoms of PVD.

## Key points

- What is the incidence of acute complications of intravitreal injections that result in urgent follow-up visits causing significant burden to both patient and physician?
- In this retrospective cohort study, 73,286 intravitreal injections were examined and 441 of these injections resulted in urgent or unscheduled follow up visits due to acute complications.
- With patient education, proper reference materials, and screening procedures urgent or unscheduled follow up visits for acute complications could be lessened.

## Introduction

Intravitreal injections are one of the most common procedures performed by ophthalmologists in the United States. With 5.9 million completed in 2016, this number is rising each year as retina specialists can perform as many as 50 per day [1]. These injections are utilized to administer critical medications into the eye that treat diseases including but not limited to: proliferative diabetic retinopathy [2], neovascular age-related macular degeneration [3], neovascular glaucoma [4], retinal vein occlusions [5], intraocular tumors [6], and endophthalmitis [7]. Some considerations must be made before an ophthalmologist can administer an intravitreal injection. Some of these considerations include intraocular pressure increase following prior intravitreal injections [8], history of ocular hypertension [9], and presence of active external infection or blepharitis [10]. Endophthalmitis is one of the most notable threats to visual acuity and has been researched extensively. Previous studies, including a meta-analysis have found that the incidence rate of endophthalmitis after intravitreal injection ranges from 0.015 to 0.08% [11–13]. There are few previous studies that focus on less severe complications such as subconjunctival hemorrhage or intraocular inflammation. One study found that 10% of patients developed subconjunctival hemorrhage [14], and 0.09% to 2.9% of patients developed intraocular inflammation [15]. However, these studies were limited by sample size, medications analyzed, and characterization of the full scope of complications encountered.

Risk cannot be completely negated when administering intravitreal injections. A recent study completed in 2020 by the Cleveland Clinic found that of 44,734 injections administered between 2012 and 2016, 841 resulted in a complication [15]. Although 1.9% is a low complication rate, the volume of patients injected creates a substantial number of patients affected. Due to this, clinicians will likely confront a complication stemming from intravitreal injections.

This study intends to evaluate the incidence of acute complications following intravitreal injections which occurred within seven days of injection. Comprising the largest and most recent study to date, we examined 73,286 injections over a 2-year period from 8/2018 to 8/2020 by 15 retinal specialists of a single private practice in Ohio.

## Methods

A retrospective cohort study was performed at a private retinal practice in Ohio over a 2-year period from 8/2018 to 8/2020. Data was obtained from the practice management software database, Current Procedural Terminology (CPT) codes were searched for all patients who received an IVI during the study period. This study was found to not constitute human subjects research by the IRB. The search was further refined to include only those with unscheduled or urgent visits within 7 days of injection. Data collected included: age, gender, affected eye, medication injected, diagnostic reason for injection, diagnosis at urgent follow up, length between date of injection and urgent follow-up, and type of anesthesia used during injection.

## Results

A total of 73,286 injections were performed by 15 retinal specialists during the study period, with 441 injections (n = 441) resulting in urgent follow-up visits (0.60%). Demographics of the groups included mean age at 72.1 ± 30.4 years, 187 male patients (42.4%) and 254 female patients (57.6%), and eye involved (OD: 51.5%, OS: 48.5%) (Table 1).

**Table 1:** Demographics of Retina Associates of Cleveland Inc. Patients Seen for Urgent/Unscheduled Visits Within 7 Days of an Intravitreal Injection

N = 441		
Demographic	Frequency	Percentage
Gender		
Male	187	42.4
Female	254	57.6
Mean age	72.1 years	
Affected eye		
OD	227	51.5
OS	214	48.5

Diagnostic reason for injection of the patients who required urgent follow-up included: wet age-related macular degeneration (52.6%), diabetic macular edema (16.1%), diabetic retinopathy (7%), vitreous hemorrhage (6.8%), branch retinal vein occlusion/central retinal vein occlusion (12.7%), neovascular glaucoma (3.2%), uveitis 0.23%), myopic choroidal neovascularization (0.68%), and persistent pseudophakic cystoid macular edema (0.23%) (Table 2). Diagnosis at time of urgent follow-up visit included: blurred vision (37.2% of urgent follow-up (UFU), 0.22% of total injections (TI)), Flashes/floaters/posterior vitreous detachment (12.5% UFU, 0.075% TI), pain ( 9.5% UFU, 0.057% TI), corneal abrasion ( 9.8% UFU, 0.058% TI), subconjunctival hemorrhage (7.5% UFU, 0.045% TI), corneal dryness/foreign body sensation (6.6% UFU, 0.041% TI), endophthalmitis (4.5% UFU, 0.027% TI), vitreous hemorrhage (4.1% UFU, 0.025% TI), iritis/uveitis (4.1% UFU, 0.015% TI), elevated IOP (1.6% UFU, 0.010% TI), choroidal neovascular membrane (0.9% UFU, 0.0054% TI), traumatic cataract (0.45% UFU, 0.003% TI), and miscellaneous (2.0% UFU, 0.012% TI) (Table 3). Medications utilized for IVI are listed as follows: Aflibercept (42.9% UFU, 60.3% TI), Ranibizumab (7.9% UFU, 22.4% TI), Bevacizumab (37.4% UFU, 13.4% TI), Dexamethasone intravitreal implant (6.8% UFU, 2.0% TI), Triamcinolone acetonide (2.3% UFU, 1.6% TI), Brolucizumab (2.7% UFU, 1.59% TI), Fluocinolone acetonide intravitreal implant 0.19 mg (0.0% UFU, 0.2% TI), Fluocinolone acetonide intravitreal implant 0.18 mg (0.0% UFU, 0.03% TI) (Tables 4 and 5). Type of anesthesia used during initial injection of the patients with UFU included: lidocaine drops (86.2%), subconjunctival lidocaine injection (12.5%) and lidocaine gel (1.4%) (Table 6). The length in days between date of injection and urgent follow-up displayed a mean of 3.96 ± 2.14 days.

**Table 2:** Underlying Diagnosis for Patients Seen for Urgent/Unscheduled Visits Within 7 Days of an Intravitreal Injection

N = 441		
Diagnostic Rationale for Injection	Frequency	Percentage
Wet Macular Degeneration	232	52.6
Diabetic Macular Edema	71	16.1
Diabetic Retinopathy	31	7
Vitreous Hemorrhage	30	6.8
Branch/Central Retinal Vein Occlusion	56	12.7
Neovascular Glaucoma	14	3.2
Uveitis	1	0.23
Myopic Choroidal Neovascularization	3	0.68
Persistent Pseudophakic Cystoid Macular Edema	2	0.45
Ruptured Retinal Macroaneurysm	1	0.23

**Table 3:** Diagnosis for Reason of Patients Seen for Urgent/Unscheduled Follow-Up Visit Within 7 Days of an Intravitreal Injection

N = 441			
Diagnosis at urgent follow up	Frequency	Percentage UFU	Percentage total
Blurred Vision	164	37.2	0.22
Flashes/Floaters/PVD	55	12.5	0.075
Pain	42	9.5	0.057
Corneal Abrasion	43	9.8	0.058
Subconjunctival Hemorrhage	33	7.5	0.045
Corneal Dryness/Foreign Body Sensation	30	6.6	0.041
Endophthalmitis	20	4.5	0.027
Vitreous Hemorrhage	18	4.1	0.025
Iritis/Uveitis	11	2.5	0.015
Miscellaneous	9	2.0	0.012
Elevated IOP	7	1.6	0.010
Choroidal Neovascular Membrane	4	0.9	0.0054
Retinal Detachment/Tear	4	0.9	0.0054
Traumatic Cataract	2	0.45	0.003

**Table 4:** Medications Administered to Patients Over Two-Year Study Period

N = 73,286		
Medication	Frequency	Percentage
Aflibercept	44,191	60.3
Ranibizumab	16,416	22.4
Bevacizumab	9,820	13.4
Dexamethasone intravitreal implant	1,466	2
Triamcinolone acetonide	1,173	1.6
Brolucizumab	1,165	1.59
Fluocinolone acetonide intravitreal implant .19 mg	147	0.2
Fluocinolone acetonide intravitreal implant .18 mg	22	0.03

**Table 5:** Medications Administered to Patients Seen for Urgent/Unscheduled Follow-Up Visit Within 7 Days of an Intravitreal Injection

N = 441		
Medication	Frequency UFU	Percentage UFU
Aflibercept	189	42.9
Ranibizumab	35	7.9
Bevacizumab	165	37.4
Dexamethasone intravitreal implant	30	6.8
Triamcinolone acetonide	10	2.3
Brolucizumab	12	2.7
Fluocinolone acetonide intravitreal implant .19 mg	0	0
Fluocinolone acetonide intravitreal implant .18 mg	0	0

**Table 6:** Type of Anesthesia Administered to Patients Seen for Urgent/Unscheduled Visits Within 7 Days of An Intravitreal Injection

N = 441		
Type of Anesthesia	Frequency	Percentage
Lidocaine drops	380	86.2
Subconjunctival lidocaine	55	12.5
Lidocaine gel	6	1.4

## Discussion

As IVI rates are increasing, ophthalmologists will inevitably encounter an acute complication that occurs due to an IVI. Based on the data collected, we determined that 0.60% of IVI resulted in an urgent follow-up visit. We found that the most common diagnoses at an urgent follow-up visit included: blurred vision in 164 (37.2%) patients, flashes/floaters/posterior vitreous detachment in 55 (12.5%) patients, corneal abrasion in 43 (9.8%) patients, pain in 42 (9.5%) patients, and subconjunctival hemorrhage in 33 (7.5%) patients. These diagnoses, while distressing to the patient, are not serious visual acuity threatening complications. Diagnoses that contribute to significant morbidity were exceedingly rare overall: endophthalmitis in 20 (4.5%) patients, vitreous hemorrhage in 18 (4.1%) patients, iritis/uveitis in 11 (2.5%) patients, elevated IOP in 7 (1.6%) patients, retinal detachment/tear in 4 (0.9%) patients and traumatic cataract in 2 (0.45%) patients. These complications comprise a smaller proportion of urgent follow up visits compared to benign diagnoses. Bevacizumab usage correlated to a larger proportion of patients seeking urgent follow-up visits. Bevacizumab was injected into 9,820 (13.4%) patients and accounted for 165 (37.4%) of urgent follow-up visits. The reason for this is unknown and may necessitate further investigation. The remaining injected medications used before urgent follow-up were more proportional to their total injections over the two-year study period.

To reduce the incidence of urgent follow-up visits after IVIs is difficult, due to the patient’s ability to adequately discern a benign symptom from a more ominous one. In addition to this, many symptoms may overlap with more serious conditions. For example, the most common symptom of blurred vision, may be benign or signal a vision threatening complication. However, in most cases of blurred vision, one study found that visual acuity returns to baseline [16].

Adequate patient education on what symptoms to expect after IVI and which symptoms would require an urgent follow-up with their ophthalmologist can be an important intervention to limit unscheduled follow-ups. Additionally, the patient population who received IVIs were at an average of 70 or more years old. Education within an elderly population, potentially with comorbid conditions, presents an additional challenge, which would require additional time and easily digestible materials for the patient to rely on. Two complications that can be targeted to reduce the incidence of urgent follow-up visits are subconjunctival hemorrhage and corneal dryness/foreign body sensation. These complications can be more easily discerned by patients, and with proper education and reference materials, patients may be able to handle these complications at home. Subconjunctival hemorrhage, while may appear impressive and serious, is benign and will dissipate over time [17]. Reference materials including images of subconjunctival hemorrhage, as well as what to look for in a more serious bleed, could limit urgent follow up visits. Corneal dryness/foreign body sensation may be limited by instructing patients to utilize artificial tears liberally for the days following an IVI [15, 18, 19]. Screening procedures could also be a method employed by ophthalmic practices to sift through benign complications of IVI. Patient provided images, telehealth visits with non-physician medical staff, and phone calls with proper staff education on what would necessitate an urgent follow-visit, and what may require reassurance, may decrease urgent follow-up load. Though these screening procedures should always err on the side of caution, as patient reporting and staff interpretation could lead to missed opportunities to treat a patient with potentially severe consequences.

This study with the most recent data and largest sample size to date of 73,286 injections will benefit clinicians that administer IVIs as it will help them anticipate what to expect in patients returning after intravitreal injection and shows trends in what acute pathologies are associated with intravitreal injections. Our study demonstrates that patients returning urgently have a 1 in 20 chance of endophthalmitis, a vision-threatening complication. The majority of patients do not have complications that necessitate a visit, and of those who come in for an urgent follow-up visit, the majority do not have serious complications. Patient education, screening, and telehealth appointments with medical staff are methods that may be employed to reduce the burden of urgent follow-up visits.

Limitations of the study include being a retrospective study involving only one private practice in a single state. Secondly, the low rate of medical complications makes statistical analysis difficult for safety. Thirdly, sub-tenon anesthesia is known to cause complications due to the invasiveness of directly injecting medication into the sub-tenon’s space. Therefore, this could have led to higher rates of complication as a result of the anesthesia provided and not due to the intravitreal injection [20]. Also, patient self-reporting may have led to underreported complications. Lastly, injection site and sterile technique used was not tabulated and may or may not have impacted the results [21, 22].

Further studies may be indicated to explore the trends between medications and rate of acute complications following intravitreal injections. The contents of the intravitreal medication, implant versus liquid, necessitate further exploration due to the differences in medication delivery as certain types of delivery devices could impact the rates of complication. More specifically, intravitreal implants and injected solutions may have disparate complication profiles as delivery systems for implants differ from those of injectable solutions. Future studies may be focused on elucidating the differences in complications between these types of injections, or amongst implant delivery systems. Trends between physician competency, skill, and surgical expertise associated with rate of acute complications following intravitreal injections is also of interest. In addition to this, the severity of symptoms among those with acute complications following intravitreal injection may also be a point of interest.

## Conclusion

Intravitreal injections resulted in an incidence of 0.60% urgent unscheduled follow-up visits within 7 days of injection in this large retinal specialty practice. Blurred vision and symptoms of PVD were the most common causes of urgent visits.

## Data Availability

Data and all other materials, deemed relevant, are available upon request by the publisher.
